# Correlation of Flexor Pollicis Longus Tendon Status by Ultrasonography with Plate Position on Radiographs Following Volar Plate Fixation of Distal Radius Fractures with Pronator Quadratus Repair

**DOI:** 10.1007/s43465-021-00369-7

**Published:** 2021-02-19

**Authors:** Anil. K. Bhat, Ashwath. M. Acharya, Prajwal P. Mane, Lakshmikanth. H. Karegowda

**Affiliations:** 1 Hand and Microsurgery unit, Department of Orthopaedics, Kasturba Medical College, Manipal, Manipal Academy of Higher Education, Manipal, Karnataka, India; 2 Department of Radiology, Kasturba Medical College, Manipal, Manipal Academy of Higher Education, Manipal, Karntaka, India; 3 Department of Orthopaedics, Kasturba Medical College, Mangalore, Manipal Academy Of Higher Education, Manipal, Karnataka India

**Keywords:** Distal radius fracture, Flexor pollicis longus tendon attrition, Attritional changes, Pronator quadratus atrophy, Pronator quadratus repair, Plate position

## Abstract

**Background:**

Purpose was to correlate flexor pollicis longus tendon (FPL) attrition using Ultrasonography with plate position on radiographs following volar locked compression plate fixation (LCP) in patients who have undergone pronator quadratus (PQ) repair for distal radius fractures.

**Methods:**

Status of flexor pollicis longus tendon was analyzed by ultrasonography in patients who underwent volar locked compression plating with pronator quadratus repair at a minimum of one year follow up. Soong’s criteria was used to assess the plate position and then correlated the ultrasonography findings of flexor pollicis longus.

**Results:**

There were 33 patients included in our study, of which 15 belonged to Soong’s grade zero, 10 were grade one and eight were grade two. Flexor pollicis longus attrition was noted in all cases with grade two plating.

**Conclusion:**

Pronator quadratus repair may not prevent attritional changes in higher grades of Soong’s, hence follow up may be required in these patients to identify attritional changes and early implant removal to prevent complications.

## Introduction

Distal radius fracture is one of the commonest fractures treated by orthopedic surgeons and accounts for 2.5% of all cases presenting at the emergency room [1]. Treatment strategies for these fractures range from conservative management with cast, percutaneous k wire fixation, external fixator and open reduction and internal fixation with various designs of plate [2]. The appropriate treatment depends on fracture pattern and associated soft tissue condition, age of the patient and other variables like fracture comminution, pattern and displacement. The volar locked compression plate (LCP) has gained popularity in the recent years and is used in wide varieties of fracture pattern. This method enables good functional results with better fragment stability and lesser complication rates when compared to other procedures [1]. Nevertheless, volar locked compression plating are associated with complications like infection, stiffness and rarely tendon ruptures or attrition due to very distal plate position which may irritate these tendons [3]. To access the fracture site and better plate position, the pronator quadratus tendon has to be dissected from its distal and radial side [2]. Many surgeons support pronator quadratus (PQ) repair post plating to facilitate the gliding of flexor tendons and minimize the irritation from the plate [4–6]. Secondary benefits include improved pronation, grip and pinch strength postoperatively [7, 8]. However a recent systematic analysis done by Marjolein et al. did not show any difference in patients with and without PQ repair in terms of improved grip and pinch strength [1, 9]. In addition, many surgeons debate that tight closure of PQ leads to ischemic contracture of the muscle which may lead to decreased supination and pronation. The flexor tendon attrition and ruptures appears to be related to inappropriate plate position, where crossing the water shed line may increase the possibility of tendon irritation [10]. This study was undertaken to look for flexor pollicis longus (FPL) tendon attrition or rupture by ultrasonography (USG) and correlate the same with radiographs for plate position following volar locked compression plating in patients who had undergone PQ repair for distal radius fractures. We also noted the status of PQ repair. The objective was to know whether the information provided by the USG would be useful to a surgeon for deciding early plate removal.

## Materials and Methods

### Surgical Steps

This was a prospective study which included 33 patients operated for distal radius fracture. The approval was taken from the institutional review board (IRB) and informed consent was taken from each patient. The inclusion criteria were as follows, all patients with closed or Gustilo-Anderson’s open type 1 distal radius fractures operated with volar locking plate with minimum one year follow up. Only those cases where PQ could be repaired to cover the distal edge of the plate were included in this study. The exclusion criteria were distal radius fractures with open type 2/type 3 open wounds and in those cases where PQ was damaged and PQ repair could not be achieved. The fractures were classified as per the AO trauma classification of distal radius fractures. The extra-articular fractures were evaluated for stability of fracture pattern as per Jupiter’s criteria and were decided for operative management [11].

All Surgeries were performed under a tourniquet control over a hand table. All cases were operated by a single surgeon trained in hand and microsurgery. Modified Henry’s approach was used in all cases and the pronator quadratus was reflected with an “L” shaped radial sided flap. In 19 cases, Synthes LCP Distal Radius Plate System (DePuy Synthes, Oberdorf, Switzerland) and in 14 cases KLS Martin Distal Radius Plate System (KLS Martin, Friedberg, Germany) were used. The plate system was chosen based on fracture pattern. In very distal fracture, juxta-articular plates were used. However Rim/hook plates were not used in any of our cases. After an adequate fixation, the PQ was repaired in all cases with utmost importance given to the cover the distal end of the plate as far as possible. Post-operative all cases were immobilized with a plaster cast for six weeks followed by which rehabilitation was initiated.

**Fig. 1:**
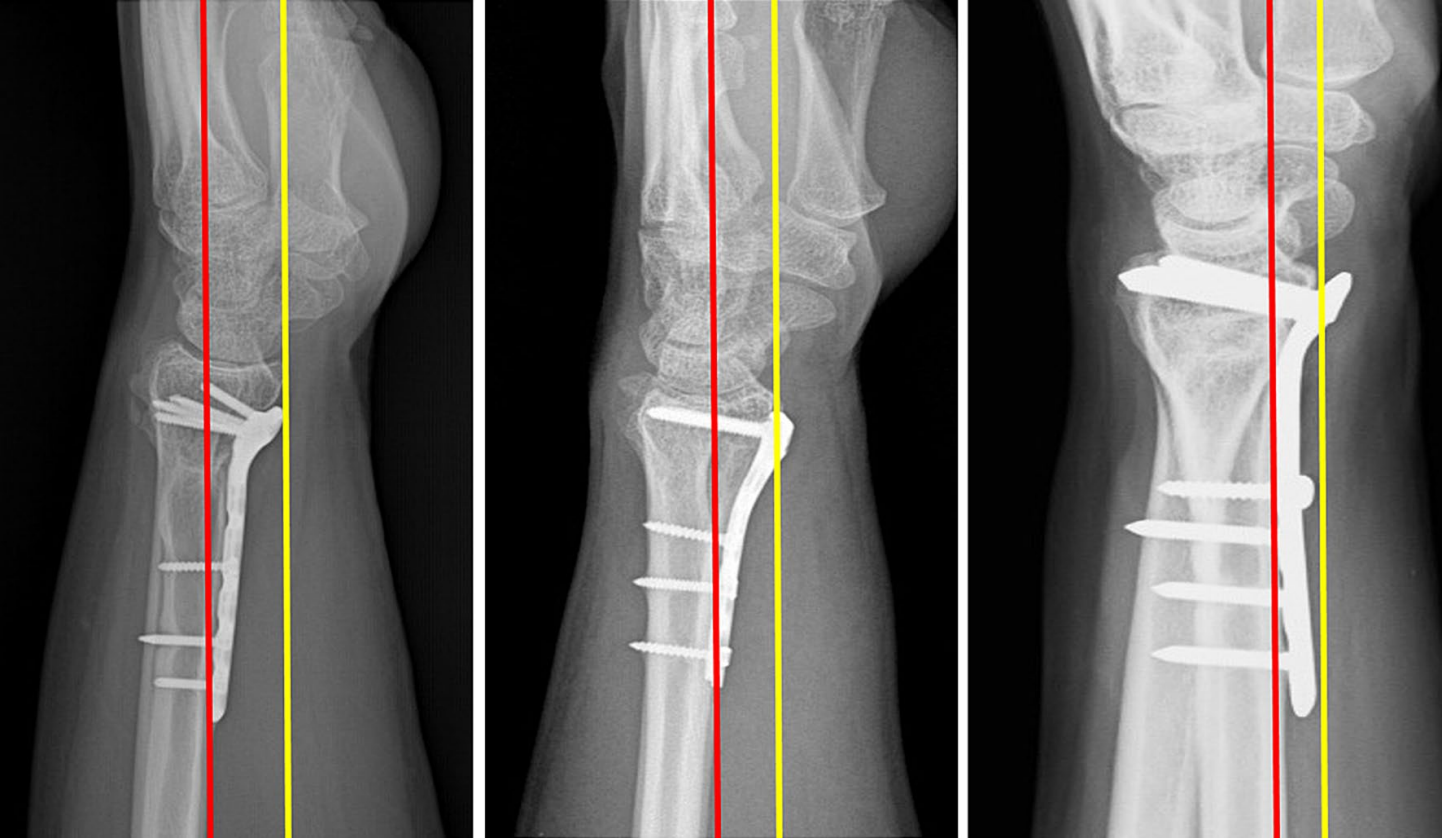
Showing the grades of plating as per Soong’s criteria. "Critical line" depicted by yellow colour line is drawn tangential to the most volar extent of the volar rim, parallel to the volar cortical bone of the radial shaft. Red line depicts the volar cortex of the radius shaft

All patients were regularly followed up at 6 weeks, 3 months, 6 months and up to a period of minimum of one year after the distal radius fracture fixation and observed for fracture healing and clinical improvement (12–36 months with mean follow up of 18 months). At the end of one year follow up, standard posterior-anterior and lateral radiographs were taken to assess the plate position according to the Soong’s criteria [10]. The true lateral radiograph was taken with forearm in midprone position and wrist in neutral position such that on drawing a straight line, axes of radius, lunate, capitate and third metacarpal all are in same line or within 10 degree coaxial [12]. On a true lateral radiograph of wrist, a "critical line" was drawn tangential to the most volar extent of the volar rim, parallel to the volar cortical bone of the radial shaft. Plates that did not extend volar to the critical line were recorded as Grade 0. Plates volar to the line but proximal to the rim (such that the recess of the pronator fossa could be clearly visualized) were recorded as Grade 1. Plates directly on or beyond the rim were recorded as Grade 2 (Fig. 1). The patients then underwent an ultrasound examination. Each patient was seated with the elbow flexed, the forearm supinated, and the shoulder in neutral position for ultrasound examination.

Ultrasonography (USG) was done by a dedicated musculoskeletal radiologist to look for any attritional changes like thinning of tendon, fibrillation changes, intra-tendinous changes and complete or incomplete tears in the FPL tendon. The status of PQ repair was also noted. The scan was performed using a high resolution L17-5 linear array transducer (17–5 MHz extended frequency range) on Philips Epic 5 ultrasound system (Philips, Amsterdam, Netherlands). The atrophic changes in FPL tendons on the operated side was detected by measuring their thickness at the level of volar lip of distal end of radius and comparing them with the contralateral side. Attritional changes were defined by the presence of thinning of the tendon, fibrillation changes, focal changes within the tendon substance or rupture of the FPL tendon. Usage of high frequency transducer ensured us in identifying the mill imetric difference in the thicknesses of the tendons. Similarly changes in PQ muscle was looked upon for any atrophic or attritional changes at the level of center of the muscle in the longitudinal axis on the operated side and compared with the opposite side. We then correlated the USG findings of FPL with plate position.

## Results

There were 33 patients with a minimum of one year follow up (maximum of three years and average follow up of 18 months) following volar plating for distal radius fracture with PQ repair included in the study. There were 23 male and 10 female patients, with a mean age of 45 years (range 25–71 years). Eighteen patients had right wrist involvement while 15 had injury on the left wrist. All patients were right hand dominant. The details of patients included in the study with respect to age, occupation, AO fracture classification, type of implant used and Soong’s grading is as mentioned in Table 1

**Table 1:** Showing the demographics details of the patients along with the fracture classification and Soong’s grading

Serial number	Age	Occupation	Fracture classification (AO)	Plate model	Soong’s grading
1	67	Driver	23-B1 (partial articular)	SYNTHES LDRS	1
2	39	Housewife	23-B1 (partial articular)	KLS MARTIN	0
3	25	Student	23-C2) (complete articular)	KLS MARTIN	2
4	53	Farmer	23-A2 (extra articular)	SYNTHES LDRS	0
5	59	Farmer	23-A2 (extra articular)	KLS MARTIN	0
6	45	Teacher	23-C1 (complete articular)	SYNTHES LDRS	2
7	60	Clerk	23-A3 (extra articular)	SYNTHES LDRS	1
8	43	Banker	23-B3 (partial articular)	SYNTHES LDRS	1
9	34	Teacher	23-B2 (partial articular)	KLS MARTIN	0
10	48	Farmer	23-B3 (partial articular)	SYNTHES LDRS	1
11	31	Electrician	23-A2 (extra articular)	SYNTHES LDRS	0
12	39	Mason	23-A2 (extra articular)	KLS MARTIN	0
13	50	Clerk	23-B3 (partial articular)	SYNTHES LDRS	1
14	40	Business	23-A2 (extra articular)	KLS MARTIN	0
15	52	Farmer	23A2 (extra articular)	KLS MARTIN	0
16	71	Retired	23-B2 (partial articular)	KLS MARTIN	1
17	31	Student	23-C3 (complete articular)	SYNTHES LDRS	2
18	56	Teacher	23-C1 (complete articular)	SYNTHES LDRS	2
19	59	Banker	23-A2 (extra articular)	SYNTHES LDRS	0
20	35	Carpenter	23-C1 (complete articular)	SYNTHES LDRS	2
21	61	Farmer	23-B2 (partial articular)	KLS MARTIN	1
22	29	Mechanic	23-C3 (complete articular)	SYNTHES LDRS	2
23	52	Farmer	23-A3 (extra articular)	KLS MARTIN	0
24	49	Business	23-A2 (extra articular)	KLS MARTIN	1
25	50	Farmer	23-B3 (partial articular)	SYNTHES LDRS	0
26	56	Banker	23-A2 (extra articular)	KLS MARTIN	0
27	31	Business	23-A3 (extra articular)	KLS MARTIN	0
28	29	Student	23-C1 (complete articular)	KLS MARTIN	1
29	31	Mechanic	23-B3 (partial articular)	SYNTHES LDRS	2
30	59	Farmer	23-A3 (extra articular)	SYNTHES LDRS	0
31	48	Teacher	23-A3 (extra articular)	SYNTHES LDRS	1
32	54	Housewife	23-B3 (partial articular)	SYNTHES LDRS	2
33	26	Student	23-A2 (extra articular)	SYNTHES LDRS	0

Soong’s criteria for plate position is shown in Fig. 1. As per this criteria, 15 patients belonged to grade 0 (12 extra-articular fractures, 3 partial -articular fractures), 10 patients belonged to grade 1 (3 extra-articular fractures, 6 partial-articular fractures, one complete articular fracture) and eight patients belonged to grade 2 (2 partial-articular fractures, 6 complete articular fractures).

The association of FPL attrition and PQ atrophy with the plate position is shown in Table 2.

**Table 2:** Table showing the association of flexor pollicis longus attrition (FPL) and pronator quadratus (PQ) atrophy with the plate position

Soong’s grading of plate position	Number of patients (*n* = 33)	FPL attrition	PQ atrophy
Grade 0	15	0	5
Grade 1	10	3	4
Grade 2	8	8	5

FPL attrition was noted in all cases with Soong’s grade 2 plate position. The Fisher’s exact test showed a significant (*p* value < 0.001) for association of plate position with FPL attrition when the plate is placed distal and volar to the watershed line, Fisher's exact test showed no significant association of plate position with PQ repair (*p* = 0.584)

### FPL Changes

FPL attrition was noted in all cases with Soong’s grade 2 plate position. The Fisher’s exact test showed a significant (*p* value < 0.001) for association of plate position with FPL attrition when the plate is placed distal and volar to the watershed line. However the Fisher’s exact test showed no significant (*p* value 0.584) association of plate position with PQ atrophy (Table 2).

**Fig. 2:**
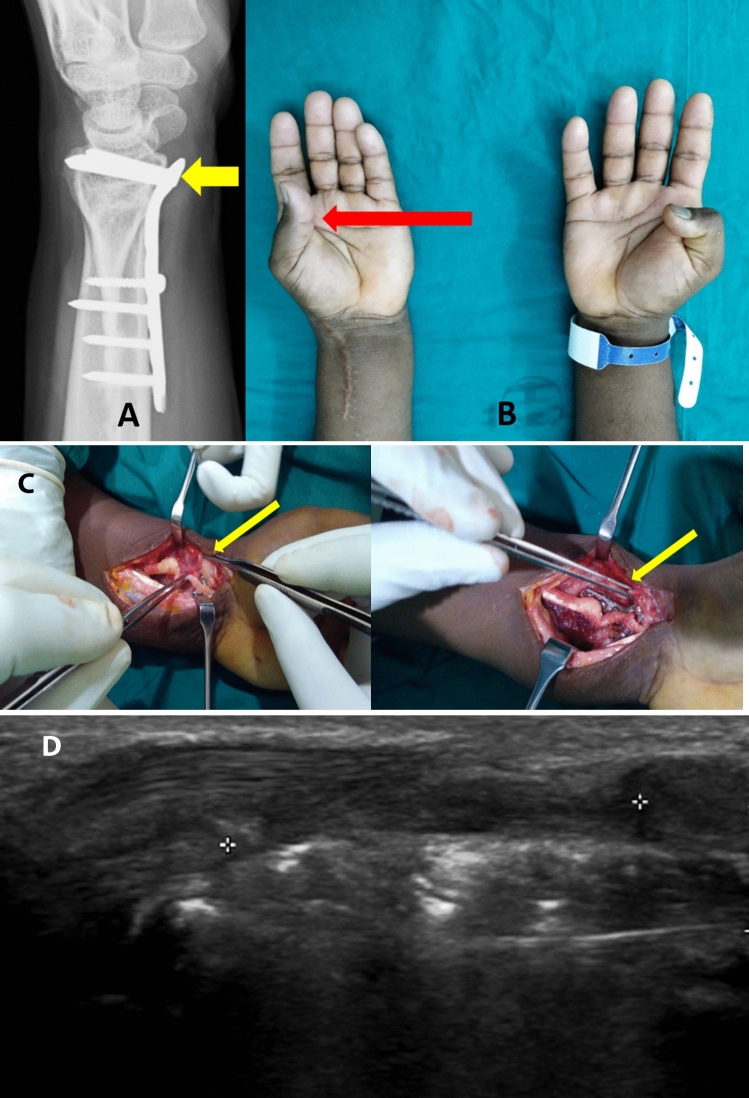
**a** Lateral radiographs of the wrist with grade 2 plate position as pointed by the yellow pointer. **b** Clinical photographs showing the inability to flex the interphalangeal joint of the thumb on left side suggestive of flexor pollicis longus injury (red pointer). **c** Intra-operative photographs showing the thinned out FPL tendon with attrition and close proximity to the distal edge of the plate as shown by the yellow pointer. **d** USG image showing the thinned out and attenuated FPL tendon with FPL tear. Gap between the torn ends shown by the white markers (+) on the image

Of the 11 patients who had FPL attrition, one patient had complete rupture of FPL and the plate position was grade 2 (Fig. 2). The average FPL tendon thickness in these above 11 cases on normal side was 3.3 mm (range of 2.1–3.3 mm) at the level of volar lip of the distal end of radius when compared to operated side which was 2.8 mm (range of 2.1–2.9 mm). Thus a difference of more than 0.5 mm between the normal and operated side showed an Odd’s ratio of greater than 1 (odd’s ratio > 1.1) which is suggestive of possibility of tendon attrition according to our study. Out of the remaining 22 cases which had no attrition of FPL tendon, 15 belonged to grade 0 plate position and seven belonged to grade 1. The average FPL tendon thickness on the normal side was 3.1 mm (range 2.7–3.2 mm) and on operated side was 2.9 mm (range 2.5–3.1 mm) and difference between these was 0.2 mm only. The overall mean of FPL thickness on the affected side was 2.93 mm and on the normal side was 3.12 mm. Paired T test showed significant association of the FPL attrition changes on the operated side when compared with normal side (*p* < 0.05).

### Pronator Quadratus Changes

USG assessment showed intactness of repaired PQ in all the cases. PQ atrophy was assessed based on the decrease in the thickness of PQ muscle on the operated side versus the thickness of the muscle on the contralateral normal side. There were 14 patients with PQ atrophy of which five each belonged to grade 0 and grade 2 plate position and four belonged to grade 1 and did not show any significant correlation between atrophy and plate position. The average PQ thickness of operated side was 8.31 mm and on the normal side was 9.08 mm, a difference of 0.77 mm.

In the rest 19 patients with no PQ atrophy, 10 patients belonged to grade 0, six patients belonged to grade 1 and three patients belonged to grade2 plate position. The average PQ thickness on operated side was 8.93 mm and normal side was 9.25 mm, a difference of 0.32 mm. We did not see any correlation between the plate position and PQ muscle atrophy (Table 3).

**Table 3:** Table showing the association pronator quadratus (PQ) atrophy/no atrophy with the plate position

Soong’s grade	PQ atrophy	PQ no atrophy
Grade 0	5	10
Grade 1	4	6
Grade 2	5	3

There is no significant correlation between plate position and PQ atrophy

## Discussion

Rupture or attrition of flexor tendon can be encountered after volar plate fixation for distal radius fractures [3]. Soong et al. graded the plate position on lateral radiographs and found that Soong grade 2, which is prominence of the plate above the critical line was associated with a higher risk of tendon rupture due to repeated friction of the tendon over the prominent plate [10]. Nanno et al., using the dynamic ultrasound evaluation of the FPL tendon in 25 patients following volar plating of distal radius fracture evaluated the dynamic FPL movement 1 month before the plate removal and 1 month after the plate removal. In their study, they showed that dynamic USG can be very useful detecting early tendon attrition of FPL by identifying the friction between the FPL tendon and plate margin. Similarly Tanaka et al. evaluated 40 patients following distal radius volar plate fixation with mean follow up of 12 months with ultrasonic Doppler study and established different wave forms and their association with tendon attrition. They concluded that the presence of a spiked waveform would more likely to have FPL tendon attrition [13, 14]. However Lutsky et al. found that most of the patients who needed to undergo plate removal had Soong grade 1 prominence which theoretically had lesser possibility of tendon attrition due to proper placement of plate [15]. They concluded that Soong grading was not correlated with the need for plate removal, even though it is recommended by many hand surgeons to prevent FPL tendon rupture in patients suspected to be at risk. However there was no mention of PQ repair status in their study. In our study we have repaired PQ in all our cases but have noticed that all patients with grade 2 Soong had associated FPL attrition with or without PQ atrophy and one patient had complete FPL rupture. Even though it is desirable to use the volar locked compression plate in grade 0 or grade 1 position, the placement at times is determined by the fracture pattern as juxta-articular fractures may require a very distal plate placement. Dedicated rim plates or hook plates may not be available always and are more expensive. It can also occur due to placement of the plate very distal making the plate proud leading to tendon attrition. Hence, the fracture patterns may not always correlate with the position of plate. Repairing the pronator quadratus theoretically protects the flexor tendons against the volar plate and sharp edges of the screw heads and serves as a dynamic stabilizer of the distal radioulnar joint [2, 16, 17]. However, PQ repair may not be optimal at all times. The repair itself might not always reach up to the distal most edge of the plate [2, 18]. In high energy fractures, the PQ is often seen damaged badly and the repair becomes difficult in these cases owing to friability of the torn muscle fibers [19].

In our study, all patients had undergone PQ repair post distal radius volar plate fixation. At one year follow up, all patients even though showed intact PQ repair, 14 patients out of 33 patients had PQ atrophy. We did not have any significant correlation between the plate position and pronator quadratus atrophy and hence meticulous placement of the plate becomes utmost important for reducing attrition of the tendon. We have performed the USG at the end of one year follow up to note the changes in FPL and PQ. Based on our findings we recommend that if the plate has to be positioned very distally when a fracture pattern demands so or if the PQ repair was not adequate or if the surgeon has erred in placing the plate very proud, then the patients are to be followed up regularly with a dynamic USG examination and any early signs of FPL or other flexor tendon attrition should be noted.

Early implant removal can be advocated in these patients once the fracture shows union to prevent tendon rupture. From our study we conclude that the plate position has greater influence on FPL tendon following distal radius fracture fixation with pronator quadratus repair. Grade 2 plate position causes more damage to FPL [20]. Also PQ repair help in reducing the risk of attrition of flexor tendons whether they atrophy or not. The repair however has to be viewed in the background of the plate position and if both measures are adequate, the risk of attrition and rupture reduces.

This study has several limitations. Firstly, the sample size our study is very small, a larger sample size would have provided more information regarding the attrition and measurements. Second, all the scans were done by only one radiologist experienced in musculoskeletal imaging, hence we could not evaluate inter-observer reliability. Inspite of these limitations, we believe that USG assessment in all the cases of distal radius fracture with very distal volar locked plate fixation will help us to determine the FPL tendon status and prevent any complications.
